# A Mechanistic Beta-Binomial Probability Model for mRNA Sequencing Data

**DOI:** 10.1371/journal.pone.0157828

**Published:** 2016-06-21

**Authors:** Gregory R. Smith, Marc R. Birtwistle

**Affiliations:** Department of Pharmacology and Systems Therapeutics, Icahn School of Medicine at Mount Sinai, New York, New York, United States of America; Wageningen UR Livestock Research, NETHERLANDS

## Abstract

A main application for mRNA sequencing (mRNAseq) is determining lists of differentially-expressed genes (DEGs) between two or more conditions. Several software packages exist to produce DEGs from mRNAseq data, but they typically yield different DEGs, sometimes markedly so. The underlying probability model used to describe mRNAseq data is central to deriving DEGs, and not surprisingly most softwares use different models and assumptions to analyze mRNAseq data. Here, we propose a mechanistic justification to model mRNAseq as a binomial process, with data from technical replicates given by a binomial distribution, and data from biological replicates well-described by a beta-binomial distribution. We demonstrate good agreement of this model with two large datasets. We show that an emergent feature of the beta-binomial distribution, given parameter regimes typical for mRNAseq experiments, is the well-known quadratic polynomial scaling of variance with the mean. The so-called dispersion parameter controls this scaling, and our analysis suggests that the dispersion parameter is a continually decreasing function of the mean, as opposed to current approaches that impose an asymptotic value to the dispersion parameter at moderate mean read counts. We show how this leads to current approaches overestimating variance for moderately to highly expressed genes, which inflates false negative rates. Describing mRNAseq data with a beta-binomial distribution thus may be preferred since its parameters are relatable to the mechanistic underpinnings of the technique and may improve the consistency of DEG analysis across softwares, particularly for moderately to highly expressed genes.

## Introduction

Since the advent of the microarray around the turn of the 20^th^ century, whole transcriptome profiling has been of great importance to systems biology [1–8]. The ability to observe how every transcript in a cell population responds to, for example, treatment with a drug or a change in the expression of a gene-of-interest, gives insight into the wiring and function of biological systems. A common method for deriving biological knowledge from such perturbation experiments is to identify lists of differentially expressed transcripts or genes (DEGs) between two (or more) conditions. By analyzing the genes which show up on such lists, one can identify larger functional units such as biological processes, pathways, networks, and organelles that are involved in the response, giving clear hypotheses for further targeted experiments [9–13]. The centralized collection of most transcriptome experiments in databases such as the gene expression omnibus (GEO) and the connectivity map (CMAP) has given further insight by enabling the use of big data methods to identify general trends and connections that do not emerge from a single experiment (or even a handful) [14–16].

While the microarray was the transcriptomic workhorse in the 2000s, the advent of massively parallel sequencing has given rise to deep mRNA sequencing (mRNAseq) [17,18], an alternative way to measure the transcriptome. Like most new technologies, mRNAseq was originally much more expensive than microarrays; however, it has now become quite competitive, and in many ways a superior technical method for transcriptome profiling [19–22]. The basic premise is to isolate mRNA from a sample, PCR amplify it, and then subject it to tens-of-millions of “short” (~50–100 bp typically) sequencing reads. By aligning the resulting sequence reads with the known genome, and then counting the number of reads that align to a particular gene or transcript, one obtains a measurement of expression. One caveat of this traditional form of quantification is the inherent PCR bias that can distort the original number of transcripts in the sample. A recent method based on incorporating a short unique molecular identifier (UMI) sequence into every transcript molecule provides a new method of quantification that reduces PCR bias and thus improves linearity and precision [23–25].

Several open source software suites with associated probability models have been developed to analyze mRNAseq data and identify DEGs. The first was Cufflinks / Cuffdiff [17], which has an elegant underlying mathematical model to estimate the “fragments per kilobase of transcript length per million mapped reads” (FPKM) metric of gene expression, and a t-test based on approximate normality of the resulting FPKM estimate. Cuffdiff2 [26] more accurately estimates false discovery rates for DEGs. Using this FPKM metric, Cuffdiff2 is specialized to a transcript-resolution of gene expression, and comparison across different transcripts, but not to count based data, which we focus on here. Other widely used software suites are EdgeR [27], DESeq2 [28] and BaySeq [29], which, as opposed to the FPKM metric of Cufflinks/Cuffdiff, retains the count-based nature of mRNAseq data and describes it with a negative binomial model (also called Poisson-gamma). This probability model describes mRNAseq count data well, and was predominantly used because it is the common choice to describe count-based data that are “overdispersed” (i.e. variance that is greater than the mean) relative to the Poisson distribution (variance = mean); it is well established that mRNAseq data are overdispersed [30,31]. A recent meta-analysis found that each of these softwares can produce quite different DEGs from the same dataset, a result that is common and not entirely surprising given the different modeling and assumptions used. Further, it was shown that the intersection of DEGs from these softwares are preferred to reduce false positives, which indicates that each might benefit from improvements to the underlying probabilistic treatment of the mRNAseq data [32].

To that end, other probabilistic distributions have been examined. The beta-binomial distribution has also been explored, and it also reflects the overdispersion of the data [33,34]. DEG analysis based upon a beta-binomial distribution is now available as an option for BaySeq solely for paired data (distinct from traditional DEG analyses) [35] and in the software BBSeq [36]; however, a derivation of the mean-variance relationship inherent in the beta-binomial distribution has yet to be undertaken. Furthermore, each software, as with negative-binomial or Poisson methods, has its own specific interpretation of the probabilistic models utilized resulting in often very different selections of DEGs following analysis. This suggests the necessity of a theoretical derivation of an appropriate probabilistic distribution: a ground-up, first-principles approach to modeling the mean-variance relationship and overdispersion which, to date, has not been deeply investigated.

Here, we propose that the basic mRNAseq experimental process is mechanistically a binomial experiment: a series of *N* trials (reads) with an essentially constant probability of success for a particular transcript/gene in each trial. This gives rise to a binomial distribution for counts from technical mRNAseq replicates, with parameters that have physical interpretation. We highlight how this binomial model agrees well with literature data for technical replicates. For biological replicates, we propose that a beta-binomial distribution, where the probability of success follows a beta distribution, can describe the data, and demonstrate its fit to two large literature datasets. Given ranges of beta-binomial parameter values typical for mRNAseq experiments, a quadratic polynomial scaling between variance and mean emerges, as is consistently experimentally observed. The dispersion parameter is the quadratic coefficient that controls this scaling, and our analysis suggests that the dispersion parameter is a continually decreasing function of the mean. Surprisingly, this is different from current approaches that impose an asymptotic value on the dispersion parameter at moderate and high mean read counts. We show how this leads to overestimating variance for moderately to highly expressed genes, which inflates false negative rates in downstream DEG analysis. Because the beta-binomial model emerges from the mechanism of the mRNAseq technique, it may be preferred, and its use might not only help improve consistency in deriving DEGs, but also variance estimation for moderately to highly expressed genes.

## Methods

### Solving for the Dispersion Parameter

For each gene *i*, we assume $\sigma_{ij}^{2}=\mu_{ij}+\varphi_{i}\mu_{ij}^{2}$ and solve for *φ*_*i*_ as follows. First, we expand the right hand side of the equation:

$$\mu_{ij}+\varphi_{i}\mu_{ij}^{2}=\frac{N_{j}\alpha_{i}}{\alpha_{i}+\beta_{i}}+\varphi_{i}\frac{N_{j}^{2}\alpha_{i}^{2}}{{\left(\alpha_{i}+\beta_{i}\right)}^{2}}=\frac{N_{j}\alpha_{i}(\alpha_{i}+\beta_{i})+\varphi_{i}N_{j}^{2}\alpha_{i}^{2}}{{\left(\alpha_{i}+\beta_{i}\right)}^{2}}$$

$$=\frac{N_{j}\alpha_{i}^{2}+N_{j}\alpha_{i}\beta_{i}+\varphi_{i}N_{j}^{2}\alpha_{i}^{2}}{{\left(\alpha_{i}+\beta_{i}\right)}^{2}}$$

Including the left hand side provides the following equation:

$$\sigma_{ij}^{2}=\frac{N_{j}\alpha_{i}\beta_{i}(N_{j}+\alpha_{i}+\beta_{i})}{{\left(\alpha_{i}+\beta_{i}\right)}^{2}(1+\alpha_{i}+\beta_{i})}=\frac{N_{j}\alpha_{i}(\alpha_{i}+\beta_{i}+\varphi_{i}N_{j}\alpha_{i})}{{\left(\alpha_{i}+\beta_{i}\right)}^{2}}$$

After simplifying:

$$\frac{\beta_{i}(N_{j}+\alpha_{i}+\beta_{i})}{\left(1+\alpha_{i}+\beta_{i}\right)}=\alpha_{i}+\beta_{i}+\varphi_{i}N_{j}\alpha_{i}$$

Writing in terms of *φ*_*i*_:

$$\varphi_{i}N_{j}\alpha_{i}=\frac{\beta_{i}(N_{j}+\alpha_{i}+\beta_{i})}{\left(1+\alpha_{i}+\beta_{i}\right)}-\alpha_{i}-\beta_{i}$$

$$\varphi_{i}=\frac{\beta_{i}(N_{j}+\alpha_{i}+\beta_{i})}{(1+\alpha_{i}+\beta_{i})N_{j}\alpha_{i}}-\frac{\alpha_{i}+\beta_{i}}{N_{j}\alpha_{i}}$$

$$\varphi_{i}=\frac{\beta_{i}(N_{j}+\alpha_{i}+\beta_{i})-(\alpha_{i}+\beta_{i})(1+\alpha_{i}+\beta_{i})}{(1+\alpha_{i}+\beta_{i})N_{j}\alpha_{i}}$$

$$\varphi_{i}=\frac{\beta_{i}N_{j}+\beta_{i}\alpha_{i}+\beta_{i}^{2}-\alpha_{i}-\beta_{i}-\alpha_{i}^{2}-2\alpha_{i}\beta_{i}-\beta_{i}^{2}}{(1+\alpha_{i}+\beta_{i})N_{j}\alpha_{i}}$$

After some cancellation, this can be broken into two terms:

$$\varphi_{i}=\frac{\beta_{i}N_{j}-\alpha_{i}-\beta_{i}-\alpha_{i}^{2}-\alpha_{i}\beta_{i}}{(1+\alpha_{i}+\beta_{i})N_{j}\alpha_{i}}=\frac{\beta_{i}(N_{j}-1)}{(1+\alpha_{i}+\beta_{i})N_{j}\alpha_{i}}-\frac{(1+\alpha_{i}+\beta_{i})\alpha_{i}}{(1+\alpha_{i}+\beta_{i})N_{j}\alpha_{i}}$$

$$=\frac{\beta_{i}}{(1+\alpha_{i}+\beta_{i})\alpha_{i}}\frac{\left(N_{j}-1\right)}{N_{j}}-\frac{1}{N_{j}}$$

Since *N*_*j*_ is very large, $\frac{N_{j}-1}{N_{j}}\approx 1$ and $\frac{1}{N_{j}}\approx 0$. Therefore, we find that:

$$\varphi_{i}\approx \frac{\beta_{i}}{\alpha_{i}(1+\alpha_{i}+\beta_{i})}$$

This corroborates well with our original estimate. For *β*_*i*_ >>>*α*_*i*_, $\varphi_{i}\approx \frac{\beta_{i}}{\alpha_{i}(1+\alpha_{i}+\beta_{i})}\approx \frac{\beta_{i}}{\beta_{i}\alpha_{i}}=\frac{1}{\alpha_{i}}$.

### Downloading mRNAseq Data

UMI count data were obtained from the DToXS LINCS website (http://research.mssm.edu/pst/DToxS) on July 1^st^, 2015, from DToXS LINCS ID Raw-Data-R2015-06-30. Raw (Level 1) transcriptomic data released June 30^th^, 2015 were downloaded, and data from batch identifier SR-1 were used in this study. There were 15 control samples (with sample name prefix CTRL), but the sample CTRL.1.C1 was excluded because it showed poor correlation with the remaining 14 samples. There were six samples treated with the kinase-inhibitor Sorafenib, (SOR), but samples 1 and 3 were excluded as they had poor correlation compared to the remaining four. Gierlinski yeast data were acquired from the European Nucleotide Archive (ENA) (http://www.ebi.ac.uk/ena/data/view/ERP004763) consisting of 672 fastq files: 2 cell lines each with 48 biological replicates each with 7 technical replicates. Raw reads from the fastq files were then aligned using Bowtie [37] against the Saccharomyces cerevisiae genome removing reads with multiple alignments to the genome. Aligned reads were then sorted using Samtools [38] and converted into files of gene read counts using Bedtools [39]. We followed the author’s method for removing “bad replicates” that did not satisfy a quality score based upon median correlation coefficient, outlier fraction and median reduced *χ*^2^ of pileup depth. We corroborated their calculations and removed six WT biological replicates (21, 22, 25, 28, 34, 36) and four ∆snf2 biological replicates (6, 13, 25, 35) just as they had done. All raw data are given in S1–S4 Tables.

### Estimating Beta-Binomial Distribution Parameters

First, the integer count data in S1–S4 Tables were divided by their respective sequencing depth, which was calculated by summing the counts along a single column (sample). The resulting probability of success estimates for each gene were fit to a beta distribution using method of moments estimates for α and β as follows:

$$\hat{\alpha }=\bar{x}\left(\frac{\bar{x}(1-\bar{x})}{\bar{v}}-1\right)$$

$$\hat{\beta }=(1-\bar{x})\left(\frac{\bar{x}(1-\bar{x})}{\bar{v}}-1\right)$$

where $\bar{x}=\frac{1}{N}\sum_{i=1}^{N}X_{i}$ is the sample mean and $\bar{v}=\frac{1}{N-1}\sum_{i=1}^{N}{\left(X_{i}-\bar{x}\right)}^{2}$ is the sample variance. These *α* and *β* parameter estimates for each gene are also given in S1–S4 Tables.

### Data Normalization

We normalize the data by scaling each sample to have an equivalent sequencing depth as the sample with the maximum sequencing depth. That is, we take $\bar{N}=\text{max}(N_{j})$ and for each sample *j*, the normalized read counts are:

$${\bar{k}}_{ij}=k_{ij}\frac{\bar{N}}{N_{ij}}$$

### Estimation of Dispersion

To obtain a smooth trend of dispersion that follows the data as implied by our beta-binomial formulation, we fit an empirical quadratic polynomial to the plot of log(mean) vs log(dispersion) using the MATLAB fit tool (y = p1*x^2^+p2*x+p3). The parameter values for each data set, in order of (p1,p2,p3) are Gierlinski WT (0.06, -0.90, 0.236), Gierlinski ∆snf2 (0.04, -0.93, 0.26), LINCS Mapped Reads (0.007, -0.83, 0.57), and LINCS UMI (0.020, -0.96, 0.26).

To compare our approach of modeling dispersion with previous methods, we downloaded the R packages DESeq2, Version 1.12.2 [28], and EdgeR, Version 3.14.0 [27]. For DESeq2, we uploaded each data set and used the *estimateDispersions* command which generates three separates formulations of dispersion for each gene: *dispGeneEst* reflects the raw dispersion estimate from the data, *dispFit* represents a curve fit to the dispGeneEst data following the distribution expected by DESeq2 and lastly *dispersion* which is a modified version of *dispGeneEst* with outliers corrected to reflect the trend of *dispFit* values. For the purposes of our work, we use the *dispersion* value for each gene in each data set as that is the recommended setting by DESeq2. For EdgeR, we use the *estimateDisp* command which also generates three dispersion estimates for each gene: *common*.*dispersion* is a single value over all genes as a best estimate of global dispersion, t*rended*.*dispersion* represents a curve fit to genewise dispersion similar to DESeq2’s *dispFit*, and *tagwise*.*dispersion* is a gene-specific estimate of dispersion that is modified to reflect the value in *trended*.*dispersion* again similar to DESeq2’s *dispersion* value. For our work, we chose the *tagwise*.*dispersion* value for each gene.

### Estimation of p-values

For each method of acquiring a dispersion estimate, we calculate an estimated variance dependent upon the mean by solving the formula $\sigma_{ij}^{2}=\mu_{ij}+\varphi_{i}\mu_{ij}^{2}$ given the normalized mean read counts *μ*_*ij*_ and dispersion estimate *φ*_*i*_ for each gene *i* in each dataset. Then we conduct a Welch’s t test for the hypotheses that the UMI CTRL and SOR samples have the same mean for a given gene and that the Gierlinski WT and ∆snf2 mutant samples have the same mean for a given gene. To do this, we modified the Matlab method ttest2 to accept as input parameters an estimate for the mean and variance for each sample as opposed to the normalized read counts themselves generating a p value for each gene in each dataset. This is to show how different estimates of dispersion, and thus different estimates of variance, affect the resulting p values for each gene tested in each dataset.

## Results and Discussion

### mRNA Sequencing as a Binomial Experiment

**Fig 1:**
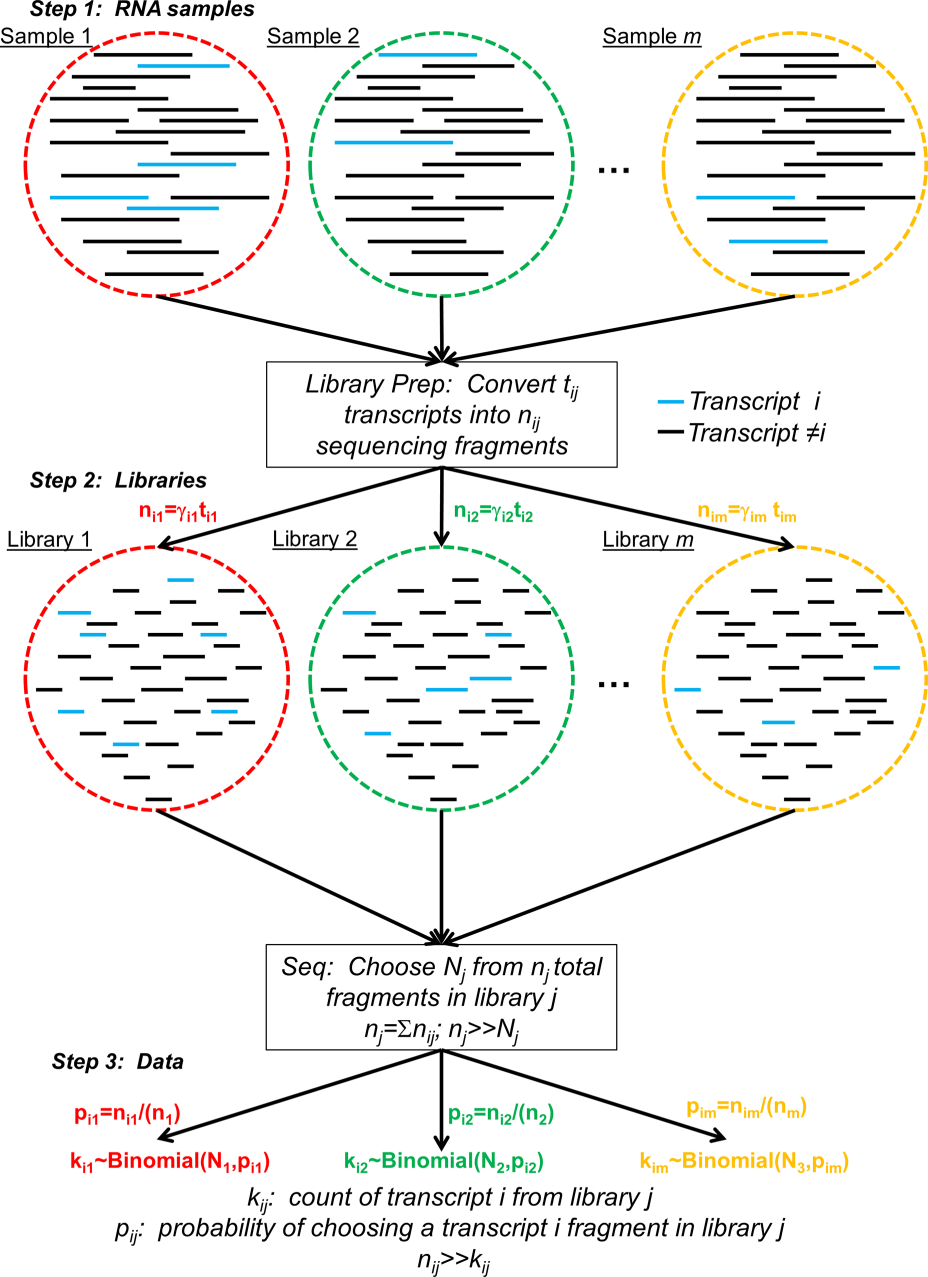
Schematic of the General mRNAseq Process. There are three main steps depicted here, from top to bottom. First is obtaining RNA samples, which contain full length transcripts. Different samples are denoted by different color circles, and transcripts by straight lines within those circles. We highlight one transcript blue to enable following it through the process. Next, library preparation converts the transcripts in each sample to a library of fragments that can be sequenced. Finally, the libraries are sequenced by choosing fragments from the library, and the number of reads that align to particular transcripts are counted for the readout of expression.

An mRNA sequencing (mRNAseq) experiment consists of three main steps (Fig 1). First is isolating mRNA from biological samples (sample index *j* ∈ {1,2,…,*m*}). Second, the mRNA samples are converted into a library that is compatible with the sequencing platform. This often includes fragmenting the original mRNA molecules, along with one or more PCR steps, into *n*_*j*_ total fragments (sometimes isolation of mRNA from total RNA is part of the library preparation). Let the number of molecules from a particular transcript *i* in the library *j* be *n*_*ij*_ = *γ*_*ij*_*t*_*ij*_, where *i* is the transcript index, *t*_*ij*_ is the original number of transcript *i* molecules in library *j*, *γ*_*j*_ *≥* 0 is the amplification factor, and $n_{j}=\sum_{i}n_{ij}$. The library is then subjected to the sequencing process, where *N*_*j*_ of the *n*_*j*_ library molecules are randomly chosen for sequencing. The number of trials *N*_*j*_ is often called the sequencing depth.

The probability of choosing a molecule for sequencing from library *j* that maps to transcript *i* is (except in relatively rare cases of capture bias)

$$p_{ij}=\frac{n_{ij}}{n_{j}}$$ (1)

Denote *p*_*ij*_ as the probability of success for transcript *i* in library *j*. If the total number of molecules in the library far exceeds the total number of reads (*n*_*j*_>>*N*_*j*_), then “taking” a fragment from the library for sequencing has negligible effect on this probability, making it essentially constant throughout the selection process. For the common Illumina platform, *n*_*j*_~10^9^ library molecules are loaded onto the instrument (e.g. ~75 μL of a 20 pM library), and a typical sequencing depth for an mRNAseq experiment is *N*_*j*_~10^7^ reads, giving *n*_*j*_>>*N*_*j*_ and essentially constant *p*_*ij*_ for all but the few most lowly expressed transcripts.

An mRNAseq experiment with library *j* can thus be cast as a series of *N*_*j*_ trials, with each trial selecting one library fragment for sequencing. We define a trial to be a success for transcript *i* if a fragment subsequently aligned to it is chosen for sequencing; the probability of success is *p*_*ij*_. This scenario, as described, is analogous to a binomial experiment [40]. Therefore, the probability of selecting *k*_*ij*_ fragments from library *j* that map to transcript *i* should follow a binomial distribution,

$$k_{ij}∼Binomial\left(N_{j},p_{ij}\right).$$ (2)

The random variable *k*_*ij*_ is often referred to as the number of uniquely mapped reads to transcript *i*, and has mean *μ* = *N*_*j*_ ⋅ *p*_*ij*_ and variance *σ*^2^ = *N*_*j*_ ⋅ *p*_*ij*_ ⋅ (1−*p*_*ij*_). In general, *p*_*ij*_ << 1 due to the large number of different expressed transcripts in a cell (typically ~10,000 [41,42] and see non-zero entries in S1 and S2 Tables). This gives *μ* = *σ*^2^ for most transcripts, as one expects from a Poisson distribution. This is in excellent agreement with data from technical replicates sequenced from the same library [22], giving direct experimental support for the notion that the mRNAseq process can be cast as a binomial experiment.

### Describing Inter-Library Variability with a Beta-Binomial Distribution

When mRNAseq experiments are performed across biological replicates which have different libraries, the probability of success for a transcript varies. Dividing the number of mapped reads for a transcript by the sequencing depth *N*_*j*_ gives an estimate of the true (inter-library) probability of success, *p*_*i*_. Because *p*_*i*_ is continuous on the unit interval (0 ≤ *p*_*i*_ ≤ 1), a potentially suitable model is a beta random variable [40], with density

$$f(p_{i})=\frac{p_{i}^{\left(\alpha_{i}-1\right)}{\left(1-p_{i}\right)}^{\left(\beta_{j}-1\right)}}{B(\alpha_{i},\beta_{i})}$$ (3)

where *B* denotes a Beta function of the first kind and *α*_i_ and *β*_*i*_ are parameters to be estimated from biological replicates. The expected value of *p*_*i*_ is

$$E[p_{i}]=\frac{\alpha_{i}}{\alpha_{i}+\beta_{i}}=\frac{E[n_{ij}]}{n_{j}}.$$ (4)

We have also used Eq 1 and the fact that the total number of library molecules is essentially constant across libraries, due to concentration normalization during loading.

When the probability of success for a binomial random variable follows a beta distribution, the resulting random variable is said to follow a beta-binomial distribution. The mean and variance of a beta-binomial distribution are, respectively [43]

$$\mu_{ij}=\frac{N_{j}\alpha_{i}}{\alpha_{i}+\beta_{i}}$$ (5)

$$\sigma_{ij}^{2}=\frac{N_{j}\alpha_{i}\beta_{i}\left(N_{j}+\alpha_{i}+\beta_{i}\right)}{{\left(\alpha_{i}+\beta_{i}\right)}^{2}\left(1+\alpha_{i}+\beta_{i}\right)}$$ (6)

As described above, predominantly, *p*_*i*_ << 1. Given Eq 4, this implies that *β*_*i*_ >> *α*_*i*_ for the majority of transcripts. Moreover, since the number of molecules in the library *n*_*j*_ is much greater than 1, it is likely that *β*_*i*_ >> 1. Given these considerations, the mean and variance reduce to

$$\mu_{ij}\approx \frac{N_{j}\alpha_{i}}{\beta_{i}}$$ (7)

$$\sigma_{ij}^{2}\approx \frac{N_{j}\alpha_{i}}{\beta_{i}}+\frac{N_{j}^{2}\alpha_{i}}{\beta_{i}^{2}}=\mu_{ij}+\frac{1}{\alpha_{i}}\mu_{ij}^{2}$$ (8)

This reveals a characteristic scaling prediction between the mean and the variance via a “dispersion parameter” 1/*α*_*i*_. Such scaling has indeed been well-described for mRNAseq experiments [27,28,30,31]. The full functional form for the dispersion parameter given a beta-binomial distribution is given in the Methods section.

### Evaluating the Beta-Binomial Model with Data from Multiple Biological Replicates

Two large mRNAseq datasets were utilized to evaluate the beta-binomial model proposed above. The first is available via the Library of Integrated Network-Based Cellular Signatures (LINCS) (see Methods—DToXS LINCS ID Raw-Data-R2015-06-30). The dataset consisted of 14 biological replicate samples (RNA isolated from independent cell batches) of PromoCell cardiomyocyte-like cells treated under control (DMSO/vehicle) conditions (S1 and S2 Tables). The sequencing libraries were prepared using unique molecular identifiers (UMI) [23–25], which allows removal of PCR biases (by experimentally estimating the γ_*ij*_ factor—see Fig 1) via quantification by UMI counts, on the level of genes. We refer to this metric as “Unique UMI Counts”. It is also possible to retain quantification by the traditional means of counting the number of reads that uniquely align to a gene. We refer to this metric as “Unique Mapped Read Counts”. The beta distribution parameters for each gene were estimated as described in Methods from the 14 biological replicates.

A second mRNAseq dataset developed by Gierlinski et al is available on the ENA archive (see Methods - project ID PRJEB5348), consisting of 48 biological replicate samples in two *S*. *cerevisiae* lines: WT and snf2 knock-out mutant [44]. The replicates underwent standard Illumina multiplexed TruSeq library preparation. Each biological replicate consists of seven technical replicates producing 336 datasets in each cell line resulting in “Unique Mapped Read Counts” (S3 and S4 Tables). As with the LINCS data, the beta distribution parameters for each gene were then estimated for each cell line as described in the Methods.

**Fig 2:**
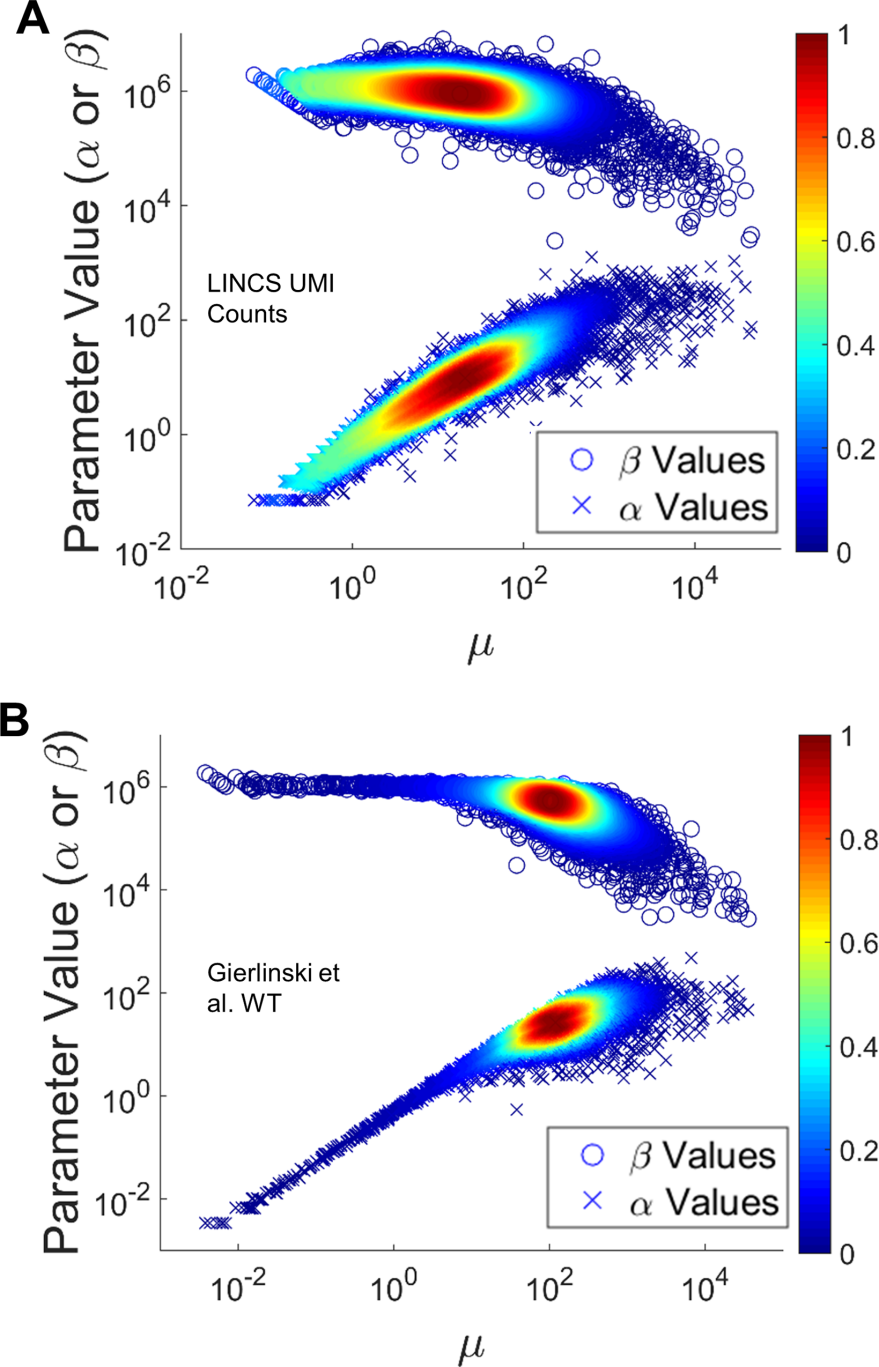
Estimated α and β values Plotted Against the Mean for each Gene. Each panel is a log-scale scatter plot of mean vs α and β over all genes for one of the following datasets tested: LINCS UMI (A) and Gierlinski WT (B). The results for the two remaining datasets are shown in S1 Fig. The x’s reflect α values and the circles reflect β values with color dependent upon the density of points in the scatter plot.

We first sought to understand the space of estimated α and β parameters for the datasets studied. Given the relationship between the beta distribution parameters and expected value for the probability of success in Eq 4, one would predict that *β*_*i*_ should remain relatively constant across genes, since most transcript types are a very small fraction of the total number of transcripts in a cell. Furthermore, we would like to evaluate the assumption above that *β*_*i*_ >> *α*_*i*_. Fig 2 shows log scale plots of α and β values plotted against the mean for two sets of count data: the LINCS UMI Counts (Fig 2A) and the Gierlinski Yeast WT Mapped Read Counts (Fig 2B). Two further sets of count data are shown in S1 Fig: the LINCS Mapped Read Counts (S1A Fig) and the Gierlinski Yeast ∆snf2 Mapped Read Counts (S1B Fig). In each panel, α values are represented by x’s and β values are represented by circles. First, we observe that β values are indeed significantly larger than α values for all genes tested. Second, β is largely invariant across the transcriptome, consistent with expectations, only slightly decreasing for genes at higher counts (relative to changes in α values). With more typical mRNAseq datasets where one might expect to have three or even fewer replicates, this result implies that a global fit of β across genes may be quite appropriate, similar to “information sharing” approaches of current softwares [27,28]. This might allow improved estimation of the dispersion parameter for each gene, particularly for those with low abundance, which is critical for estimation of variance and downstream differential expression testing [27,28,30,31]. Lastly, it is clear that the mean is largely determined by α, implying that dispersion is strongly linked to the mean.

**Fig 3:**
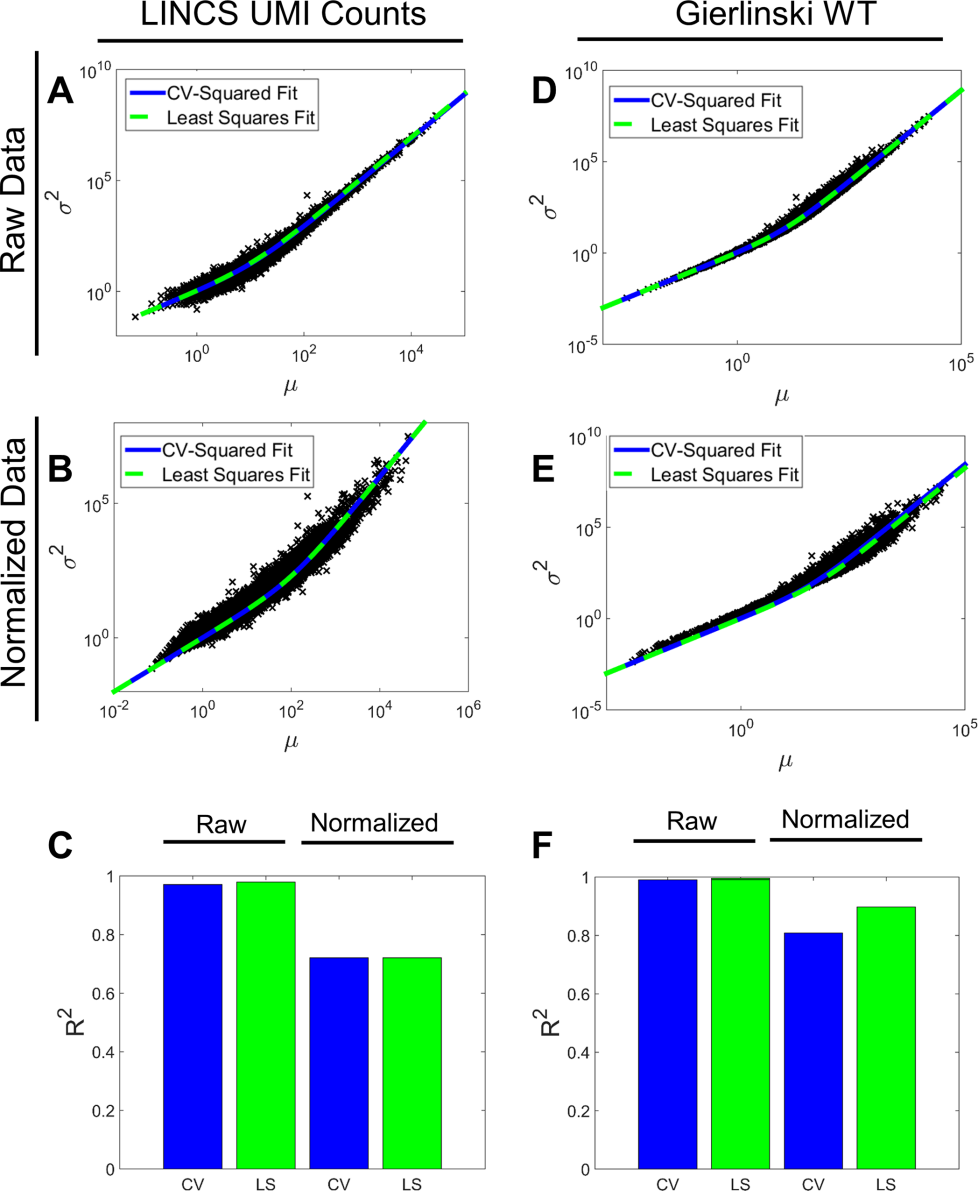
Mean-Variance Relationship for Raw and Normalized mRNAseq Data. Each column of three panels reflects one of the following datasets tested: LINCS UMI (A-B) and Gierlinski WT (D-F). The two remaining datasets are shown in S2 Fig. For each column of three panels, the first panel (A,D) shows the CV^2^ fit (solid blue line) and Least Squares fit (dashed green line) to the raw data points plotting mean vs variance (black x’s). The second panel (B,E) shows the same fits for the normalized data. The third panel (C,F) shows the respective R^2^ values for the CV^2^ and Least Squares (LS) fits for the raw and normalized data.

We next evaluated whether the beta-binomial model captured the mean-variance structure of the mRNAseq data, which is critical for determining differential expression. Here, we focus on a global gene-independent dispersion parameter, and explore gene-specific dispersion parameters subsequently. We calculated the mean and variance for each gene in each of the datasets studied and compared this to the Eq 8 prediction given a beta-binomial model and one of two global estimates for the dispersion parameter. The first estimate for dispersion is based on previous approaches: CV^2^ [27]. The second estimate utilizes least squares (LS) regression. We made this comparison for each dataset both before and after a simple scaling normalization procedure (see Methods) to account for differences in sequencing depth between samples. Table 1, Fig 3 and S2 Fig show the dispersion estimates based upon the two procedures and their respective R^2^ values. Genome-wide estimated dispersion values are very close for the LS and CV^2^ fits. However, R^2^ values are only high when fitting to the raw and not read-depth normalized data. This observation, along with Eq 8, suggests that the dispersion parameter strongly depends on the mean.

**Table 1:** CV^2^ and LS fits for the dispersion parameter *φ* for each dataset under raw and normalized conditions. R^2^ values are also included for the quality of the corresponding fit to the raw data.

Dataset	Processing	CV^2^ *φ* Fit	CV^2^ R^2^	LS *φ* Fit	LS R^2^
LINCS MR	Raw	.0700	.9218	.0898	.9687
	Normalized	.0101	.7084	.0118	.7229
LINCS UMI	Raw	.0785	.9703	.0867	.9791
	Normalized	.0099	.7205	.0098	.7205
Gier WT	Raw	.0815	.9909	.0799	.9913
	Normalized	.0271	.8083	.0173	.8974
Gier SNF2	Raw	.0684	.9794	.0606	.9961
	Normalized	.0159	.7982	.0118	.9078

### Relationship Between Dispersion and Mean

Previous work allows for gene-specific estimation of dispersion [27,28], which imposes a relationship where the gene-specific dispersion parameter asymptotes to a lower bound as mean increases. This relationship derives from the widely accepted quadratic function between variance and mean. This fixed lower bound of dispersion is sometimes called the biological squared coefficient of variation [27], and typically reaches this lower limit at moderate read counts.

**Fig 4:**
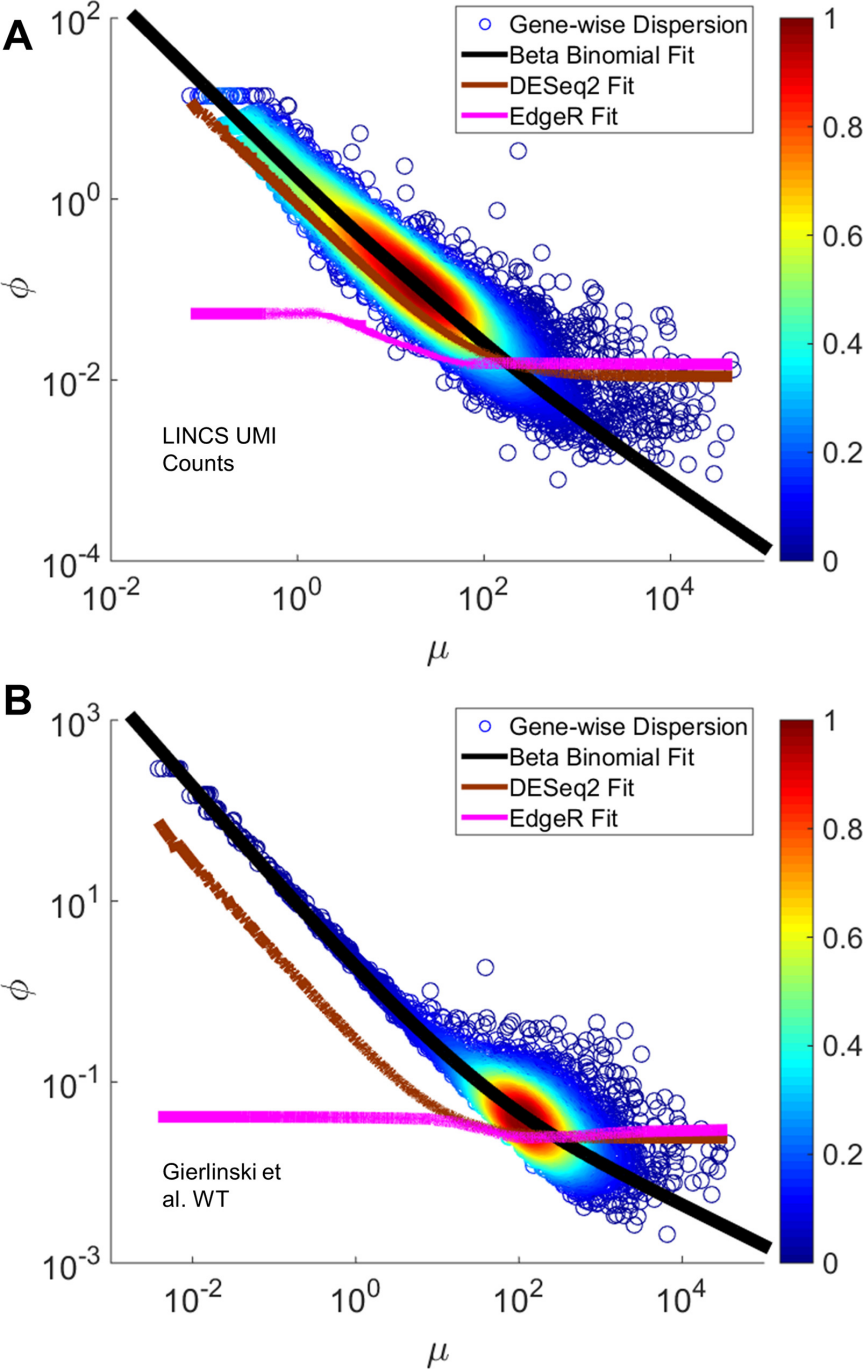
Comparing Beta-Binomial Dispersion with DESeq2 and EdgeR Dispersion Estimates. Each panel reflects one of the following datasets tested: LINCS UMI (A) and Gierlinski WT (B). The remaining two datasets are shown in S3 Fig. Each panel shows a density scatter plot of mean versus dispersion values for each gene in each sample. The black line represents our fit showing the non-asymptotic relationship between mean and variance (see Methods). The brown line shows the DESeq2 dispersion fit while the magenta line shows the EdgeR dispersion fit (see Methods).

The beta-binomial model makes a different prediction about the dependence of dispersion with the mean. Namely, because increases in mean are predominantly driven by increases in α (β is mostly constant across genes), and the dispersion parameter is essentially inversely proportional to α (Eq 8), then we expected the dispersion parameter to be smaller than that imposed by the currently used formalisms in DESeq2 and EdgeR. We compared the beta-binomial dispersion trends with those calculated by DESeq2 and EdgeR (Fig 4 and S3 Fig) for both datasets analyzed above, along with direct estimates of dispersion based on the data themselves. The results indeed displayed evidence that current estimation methods were overestimating dispersion at read counts starting at ~100 (5–10% of the genes). We conclude that a beta-binomial representation of mRNAseq data might allow for more precise estimation of gene-specific dispersion, and further that current methods might overestimate dispersion and therefore variance for moderately to highly expressed genes. This may have implications for downstream DEG analysis, since a larger variance would lead to a higher false negative rate.

### Statistical Significance of Moderately to Highly Expressed Genes

**Fig 5:**
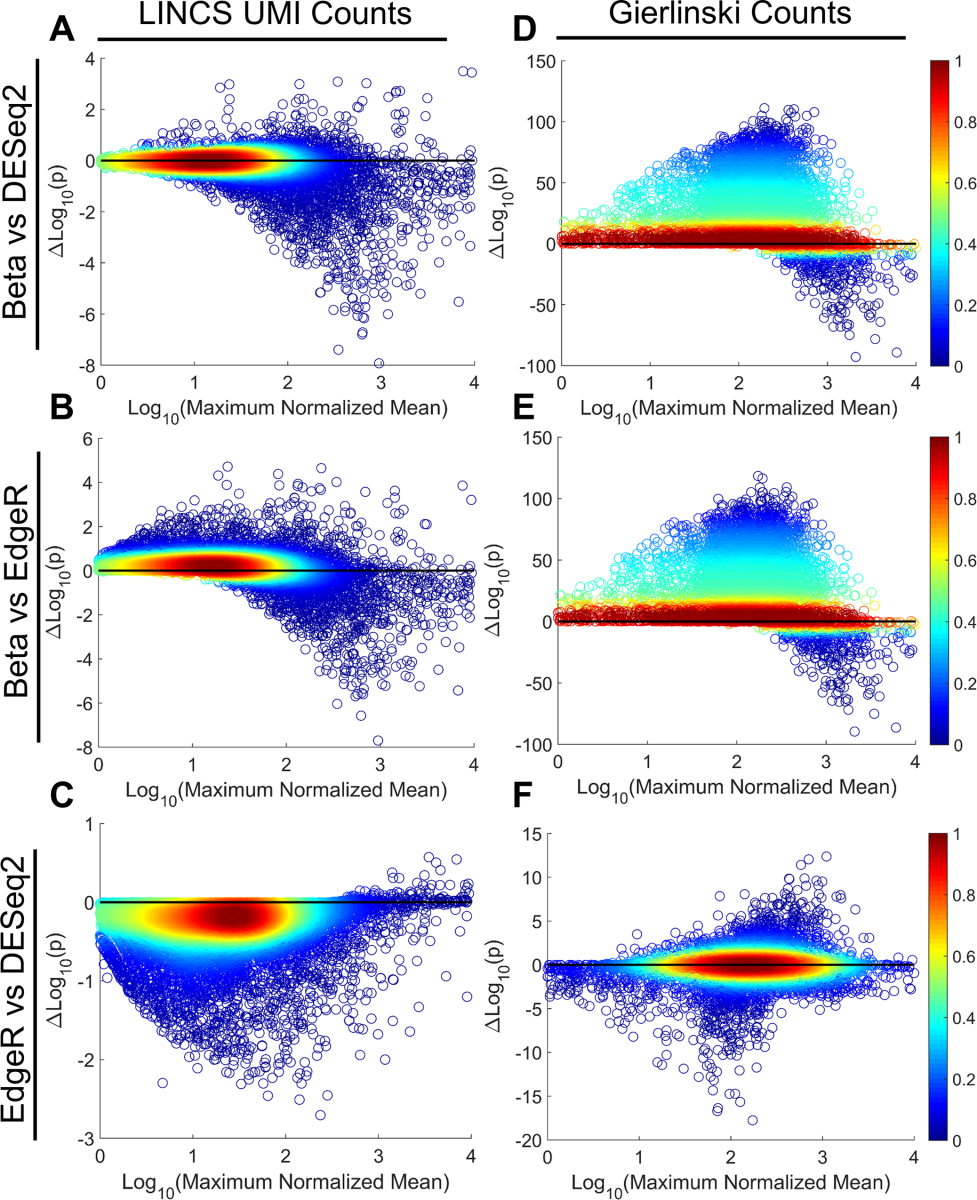
Differential p-values for Negative Binomial vs. Beta-Binomial Dispersion Methods. Each panel reflects a comparison of p-values for beta binomial-based dispersion or negative binomial-based dispersion generated from the UMI count data, CTRL vs SOR (A-C), or the Gierlinski data, WT vs ∆snf2 (D-F). Each panel is a scatter plot of the base-10 logarithm of the maximum normalized mean (maximum of the CTRL mean or SOR mean for UMI or the WT mean or ∆snf2 mean for Gierlinski) against the difference in base-10 logarithm of the corresponding p-values being compared for each gene. Color indicates density of points. The top row compares the beta binomial formulation versus DESeq2 (A,D). The second row compares beta binomial versus EdgeR (B,E). The third row compares EdgeR and DESeq2 (C,F).

To demonstrate explicitly how overestimating dispersion could lead to identification of new DEGs, we explored a comparison of treated vs. control data for the UMI data set (DMSO vs. sorafenib) and the Gierlinski dataset (WT vs. ∆snf2). We expected that for genes with moderate to high mean read counts, we would have on average higher statistical significance than current negative binomial based methods. As representative of negative binomial methods we used DESeq2 and EdgeR. Fig 5 shows precisely this prediction; as mean read counts increase, the p-values calculated for dispersion estimates of a beta-binomial model are much lower than that from typical negative binomial models. This is evidenced by a preponderance of data below zero on the difference of p-value scatter plots above 100 counts for UMI, and 200 for Gierlinski (Fig 5). This leads to several new genes being called as DEGs, which gives rise to potential new biology being uncovered. Specifically, 597 genes from the Gierlinski dataset and 1023 genes from the LINCS dataset (S5 and S6 Tables). Thus, not only does the beta binomial distribution better capture the statistical dispersion properties of mRNAseq data, but it also has biologically meaningful implications.

## Conclusions

Use of mRNAseq to measure transcriptomes is expected to increase, and derivation of DEGs is essential for extracting knowledge from such data. There is no uniform agreement on what probabilistic assumptions and models to use and as such various mRNAseq analysis softwares produce different (sometimes markedly) DEGs. This paper proposes that the mRNAseq process is inherently a binomial process, and a beta-binomial model is an appropriate choice for describing mRNAseq data. We found that current methods may be overestimating dispersion and therefore variance for moderately to highly genes, and that the beta-binomial description can correct this to achieve better sensitivity for medium to highly expressed genes. Standardizing modeling approaches can help to harmonize the DEG outputs from different softwares and thus help to increase knowledge extracted from these increasing amounts of data.

## Supporting Information

S1 Fig Estimated α and β values Plotted Against the Mean for each Gene. Continuation of Fig 2 on the two remaining datasets: LINCS Mapped Reads (A) and and Gierlinski ∆snf2 (B). The x’s reflect α values and the circles reflect β values with color dependent upon the density of points in the scatter plot.
(TIF)

S2 Fig Measuring Quality of Fit for the Beta-Binomial Model to Raw and Normalized mRNAseq Data. Continuation of Fig 3 on the two remaining datasets: LINCS Mapped Reads (A-C) and Gierlinski ∆snf2 (D-F). For each column of three panels, the first panel (A,D) shows the CV^2^ fit (solid blue line) and Least Squares fit (dashed green line) to the raw data points plotting mean vs variance (black x’s). The second panel (B,E) shows the same fits for the normalized data. The third panel (C,F) shows the respective R^2^ values for the CV^2^ and Least Squares (LS) fits for the raw and normalized data.
(TIF)

S3 Fig Comparing Beta-Binomial dispersion derivation with DESeq2 and EdgeR dispersion estimates. Each panel reflects one of the following datasets tested: LINCS Mapped Reads (A) and Gierlinski ∆snf2 (B). The black line represents our fit showing the non-asymptotic relationship between mean and variance. The brown line shows the DESeq2 dispersion fit while the magenta line shows the EdgeR dispersion fit.
(TIF)

S1 Table Raw Data and Beta Distribution Parameter Estimates for LINCS Mapped Read Data. (XLSX)

S2 Table Raw Data and Beta Distribution Parameter Estimates for LINCS UMI Data. (XLSX)

S3 Table Raw Data and Beta Distribution Parameter Estimates for Gierlinski WT Data. (XLSX)

S4 Table Raw Data and Beta Distribution Parameter Estimates for Gierlinski ∆snf2 Data. (XLSX)

S5 Table Base-10 Logarithm p-value Differences for Predicting Differential Gene Expression in Gierlinski Count Data. (XLSX)

S6 Table Base-10 Logarithm p-value Differences for Predicting Differential Gene Expression in LINCS UMI Count Data. (XLSX)
