# Supplementary figures and images for: A Mechanistic Beta-Binomial Probability Model for mRNA Sequencing Data
Source: PLoS One. 2016 Jun 21;11(6):e0157828. doi: 10.1371/journal.pone.0157828 (PMC4915702; doi:10.1371/journal.pone.0157828)

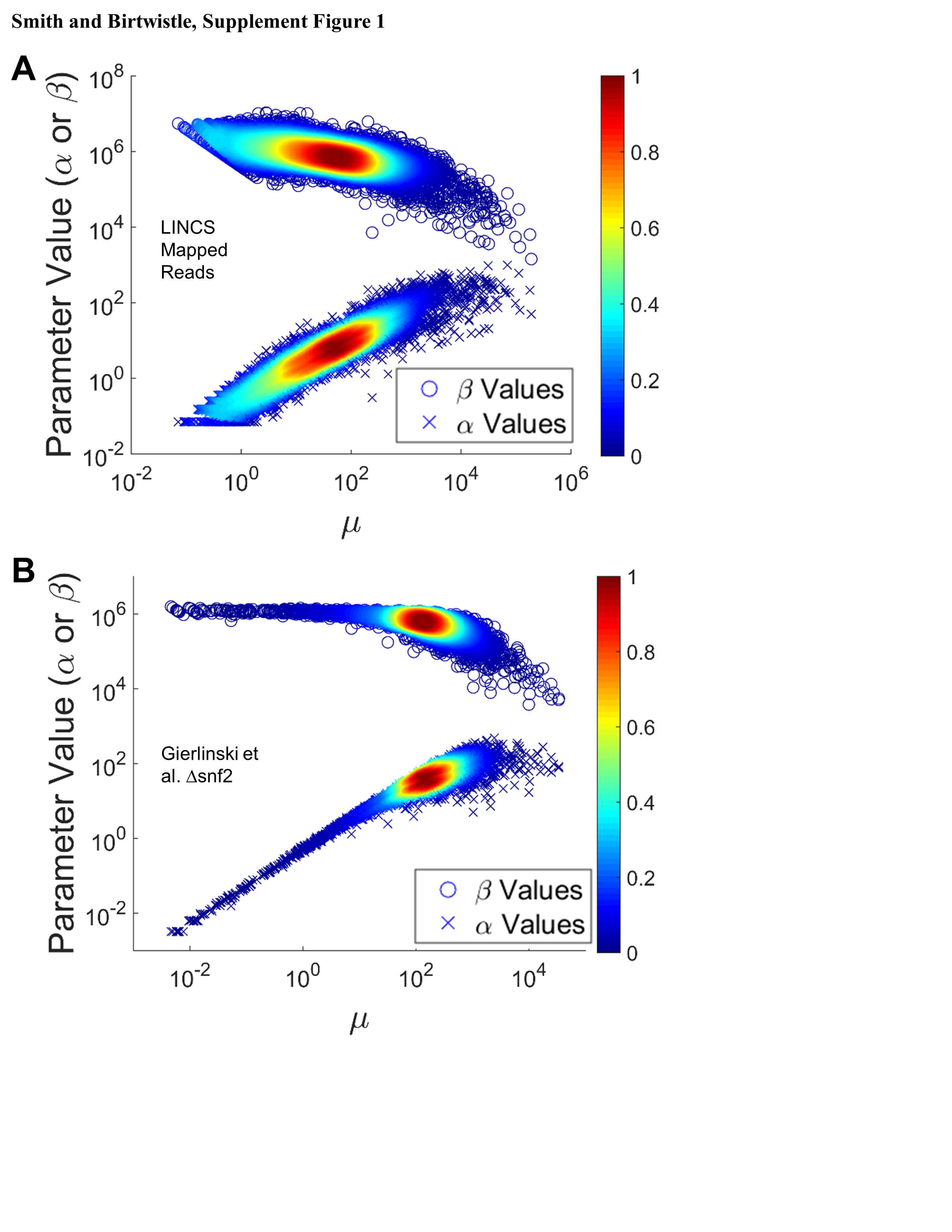

Supplement: S1 Fig — Continuation of Fig 2 on the two remaining datasets: LINCS Mapped Reads (A) and and Gierlinski ∆snf2 (B). The x’s reflect α values and the circles reflect β values with color dependent upon the density of points in the scatter plot. (TIF) [file pone.0157828.s001.tif]

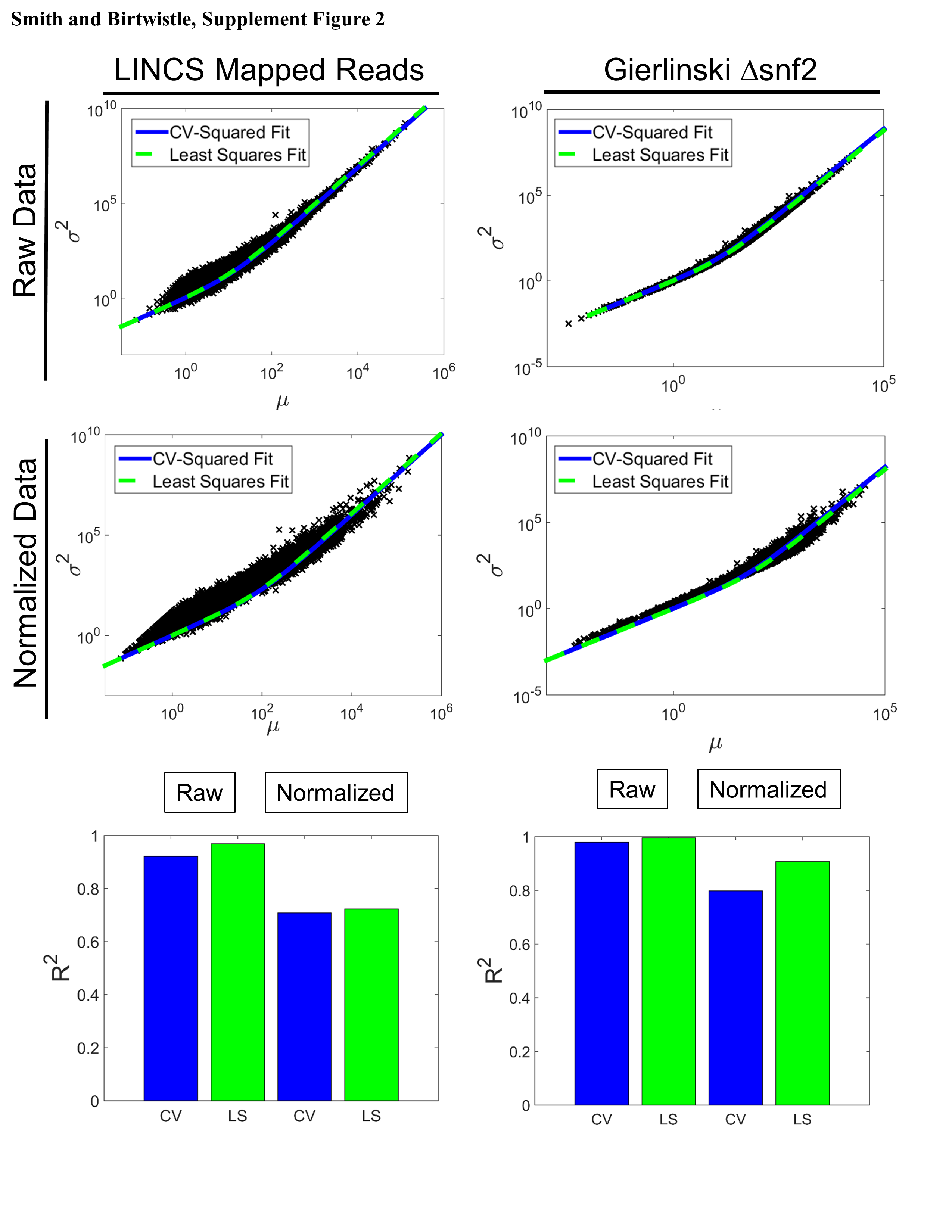

Supplement: S2 Fig — Continuation of Fig 3 on the two remaining datasets: LINCS Mapped Reads (A-C) and Gierlinski ∆snf2 (D-F). For each column of three panels, the first panel (A,D) shows the CV2 fit (solid blue line) and Least Squares fit (dashed green line) to the raw data points plotting mean vs variance (black x’s). The second panel (B,E) shows the same fits for the normalized data. The third panel (C,F) shows the respective R2 values for the CV2 and Least Squares (LS) fits for the raw and normalized data. (TIF) [file pone.0157828.s002.tif]

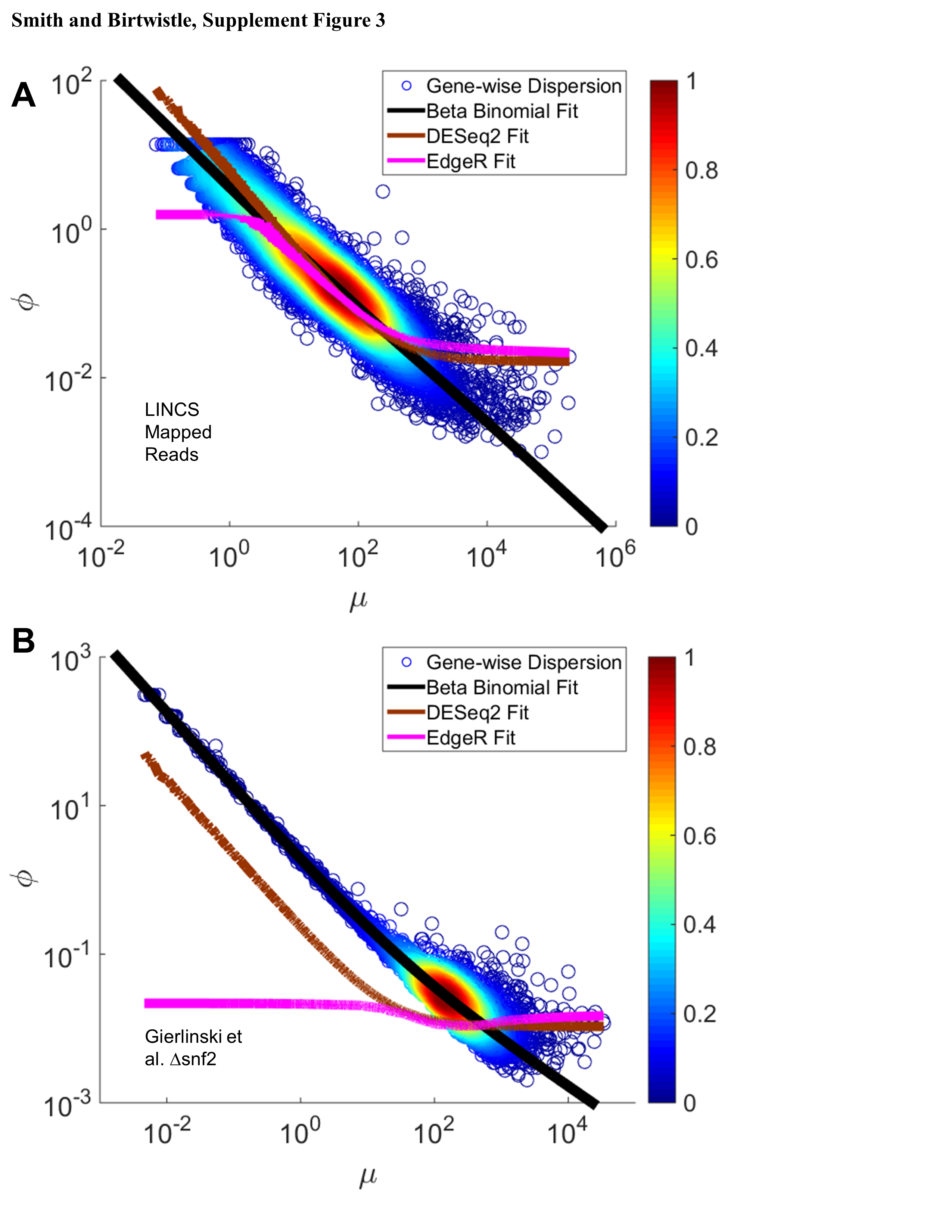

Supplement: S3 Fig — Each panel reflects one of the following datasets tested: LINCS Mapped Reads (A) and Gierlinski ∆snf2 (B). The black line represents our fit showing the non-asymptotic relationship between mean and variance. The brown line shows the DESeq2 dispersion fit while the magenta line shows the EdgeR dispersion fit. (TIF) [file pone.0157828.s003.tif]
